# Improving stroke prevention in patients with atrial fibrillation

**DOI:** 10.1186/1745-6215-14-193

**Published:** 2013-07-02

**Authors:** Derk L Arts, Ameen Abu-Hanna, Harry R Büller, Ron JG Peters, Saeid Eslami, Henk CPM van Weert

**Affiliations:** 1 Department of General Practice, Academic Medical Center, Meibergdreef 9, 1105 AZ, Amsterdam, The Netherlands; 2 Department of Medical Informatics, Academic Medical Center, Meibergdreef 9, 1105 AZ, Amsterdam, The Netherlands; 3 Department of Vascular Medicine, Academic Medical Center, Meibergdreef 9, 1105 AZ, Amsterdam, The Netherlands; 4 Department of Cardiology, Academic Medical Center, Meibergdreef 9, 1105 AZ, Amsterdam, The Netherlands; 5 Pharmaceutical Research Center, School of Pharmacy, Mashhad University of Medical Sciences, Mashhad, Iran

**Keywords:** Atrial fibrillation, Decision support, Stroke prevention, Anti-thrombotic treatment, Clinical decision support system, General practice

## Abstract

**Background:**

Patients with atrial fibrillation (AF) are at increased risk for stroke. Antithrombotic treatment reduces this risk. Antithrombotic treatment consists of either administration of oral anticoagulants (OAC) or the provision of an antiplatelet drug. International guidelines provide advice on the preferred treatment, thereby balancing the risks and benefits of OAC. However, adherence to these guidelines is reported to be as low as 50%. There is paucity in research on why adherence rates are low. Recent studies have shown decision support systems can improve guideline adherence. We investigate the use of a clinical decision support system to improve guideline adherence among general practitioners (GPs) treating patients with AF and study reasons for guideline non-adherence.

**Methods/Design:**

The study is a randomized controlled trial, which is performed among Dutch general practitioners. Initially, GPs in the vicinity of the Academic Medical Center (AMC) in Amsterdam will be included, after which other practices will be recruited. We have developed a novel decision support system that displays a list with pending messages for the on-screen medical record in real time. Messages are generated on a server that evaluates a decision rule based on the atrial fibrillation guideline of the Dutch College of General Practitioners. By interacting with the list, messages can be opened for a description and explanation, or be ignored. GPs are allocated into three groups: 1) control group; 2) intervention group A, in which messages can be ignored without justification; and 3) intervention group B, in which messages can only be ignored with justification.

Our main outcome measure is the between-group difference in the proportion of patients receiving antithrombotic prescriptions in adherence to the Dutch GP guideline for atrial fibrillation. Secondary outcomes are reasons GPs state for deviating from the guideline and the effect on guideline adherence of requiring justification when ignoring a message.

**Discussion:**

This paper describes the protocol for a cluster randomized trial to study the effects of a clinical decision support system in patients with atrial fibrillation. The system is characterized by a non-interruptive presentation and real-time messages that are updated after each relevant action the GP performs.

**Trial registration:**

This trial is registered with the Dutch Trial Register under registration number 3570.

## Background

Patients with atrial fibrillation (AF) are at high risk for stroke, with up to a fivefold risk compared to patients without AF [1]. Apart from the increased risk, stroke severity is increased [2]. Therefore, patients with AF need to be treated with antithrombotic medication to reduce stroke risk. Oral anticoagulants (OAC), such as warfarin and the new oral anticoagulants (for example, dabigatran), reduce the risk of stroke by 60%, whereas antiplatelet drugs reduce the risk by 20% [3,4]. Because OAC increases the risk of bleeding, not every patient is eligible for this treatment, as there are patients with a pre-existing increased risk of bleeding. Guidelines have been developed to help the physician decide which treatment is appropriate for a patient. These guidelines incorporate contraindications for OAC, and stroke risk stratification schemes to balance risks and benefits. Estimates of current adherence to stroke risk guidelines in the prevention of stroke in patients with atrial fibrillation is about 50% internationally, leading to both over- and undertreatment. This is true for both primary and secondary care [5-7]. Research on the reasons for these low adherence rates is lacking. We hypothesize that patient preference and medical context, lack of time and lack of overview are important drivers in this process. These are reasons that have been shown to influence guideline adherence in general in previous studies [8]. More insight into the reasons stated by physicians for non-adherence could prove valuable for future research and guideline development.

The aim of this study is to investigate the effect of a clinical decision support system (CDSS) on adherence to the guideline that recommends antithrombotic treatment for stroke prevention for patients with atrial fibrillation. Clinical decision support systems have proven to be effective interventions for improving various healthcare processes [9,10]. However, evidence for clinical improvement is sparse [9]. We hope to expand the current body of evidence for the effectiveness of CDSS with findings of increased guideline adherence and, in follow-up projects, better clinical outcomes. Furthermore, not all instances of non-adherence are undesirable. Physicians may have good clinical reasons (for example, comorbidity, social environment) to deviate from recommended treatment. Therefore, the second objective of this study is to study reasons for deviation. Lastly, we would like to evaluate whether the requirement for justification influences response to a clinical decision support system.

## Methods/Design

This study will be a cluster randomized controlled trial. Randomization at the patient level was considered, but deemed inapplicable due to the fact that one GP would have patients in both the control and intervention groups, thus leading to possible contamination bias. Randomization at the GP level was not possible because most GPs work in group practices and share patients, which would lead to a GP in the control group reading the reports of another GP who had received the intervention. This would also lead to contamination bias. It was therefore decided to randomize on the GP cluster level, where GPs treating the same patients will be regarded as a randomization unit (usually two or three GPs).

GPs will be allocated into one of the following three groups:

1. Will receive no messages (control group)

2. Will receive messages that can be ignored without justification (intervention 1)

3. Will receive messages that can only be ignored with justification (intervention 2)

### Intervention

We have developed a plugin (an extra piece of software that is not an integral part of the main system) for a single GP electronic health records system (EHRS). The CDSS plugin can be classified as active (it does not wait to be asked), non-interruptive (does not interrupt the workflow), and critiquing (provides feedback based on the current treatment scheme). The basic functionality of this decision support system is based on detecting relevant events based on triggers, evaluating the events and responding to them. Several trigger points in the GPs’ EHR have been designed. Trigger points are events that may occur in a system that then trigger an action (for instance ‘Closing a record’= > ‘send data to the server’). These triggers are:

1. opening a patient’s electronic medical record on the computer

2. adding laboratory data (for example, a new glomerular filtration rate (GFR))

3. changing medication (for example, prescribing aspirin)

4. updating the patient’s problem list (for example, registering a diagnosis of atrial fibrillation)

Whenever a trigger is activated, information from the on-screen medical record (the medical record that is currently opened on the GP’s screen) is sent to a remote server via a secure Internet connection. This information consists of recent laboratory data (12 months), active medication and a list of the patient’s diagnoses. On the remote server a clinical rule engine (CRE) evaluates the provided information using the computerized decision rule (see decision rule). The evaluation of the decision rule may lead to a response in the form of a message. This message is formatted as an XML (a file format designed to exchange data) document and sent to the CDSS plugin that has been installed on the GP’s computer. The plugin reads the XML file and evaluates the contents. It extracts the message title and displays part of the title in a thin sidebar that is attached to the right side of the GP’s screen. The GP can move the mouse cursor over the sidebar to display the full message title. He or she can then choose to either ignore the message by clicking on the ‘ignore’ button, or open the message by clicking anywhere on the message title. When the message is opened, a report is shown that displays the complete message, as well as the findings that have led to the message, and a clickable link to online background information. Upon first appearance, the message title is displayed with a red background in the sidebar, indicating the message is new. If the GP does not interact with the message, the background will become orange the next time the GP opens the electronic medical record, indicating the message has been shown before but not dealt with yet. When the GP changes the treatment plan according to the guideline, the background will turn green and the message title will be updated. This can happen without opening the message; that is, the GP might already know that he or she needs to change the treatment plan and doing so will also result in a green background and updated message without the GP having to interact with the CDSS plugin.

It is important to note that by using this procedure, real-time feedback to the GP is provided. This means that the GP will see a direct effect of every relevant action he or she performs on the screen.

When analyzing baseline data to determine the current proportion of correctly treated patients, we found that recorded diagnoses of stroke were often incomplete or inaccurate. Therefore, a single question was added to the messages of the rule engine, which asks the GP for the exact type of stroke if/when the International Classification of Primary Care (ICPC) [11] code for stroke (K90) is found on the patient’s diagnoses list. The following options will be presented to the GP: subarachnoid hemorrhage (K90.01), hemorrhagic stroke (K90.02) and ischemic stroke (K90.03). Once the question is answered, the response is saved for future use and sent to the clinical rule engine, where the entire decision rule can be evaluated.

GPs who are allocated into the third group (intervention 2) will not be able to ignore a message without providing justification. The GP is presented with several predefined options and a free text field, which will allow him or her to enter other explanations. The GP cannot continue without answering this question. When the GP tries to close the on-screen medical record without providing a reason for deviating from the advice, he or she is reminded once more of the message and asked to respond. An independent adjudication committee will value the legitimacy of the reasons provided to deviate from the advised treatment and will contact GPs when necessary.

### Decision rule

The computerized decision rule is based on the Dutch GP guideline for atrial fibrillation authored by the Dutch College of General Practitioners [12]. This guideline uses the CHADS2 score, a commonly used stroke risk stratification scheme, which is calculated by assigning 1 point each for the presence of congestive heart failure, hypertension, age 75 years or older, and diabetes mellitus and by assigning 2 points each for history of stroke or transient ischemic attack (TIA) [13].

The decision process consists of two phases:

1. The rule engine uses the decision rule to determine the recommended treatment.

2. The rule engine compares the actual treatment to the recommended treatment and generates the message according to the result.

The first phase is a two-step procedure:

1. Determine whether contraindications for OAC are present. If this is the case, antiplatelet drugs are the preferred treatment.

2. Calculate the CHADS2 score. For scores of one and lower, the preferred treatment is antiplatelet drugs; for scores of two and higher, the preferred treatment is OAC.

In the second phase, the recommended treatment is compared to the actual treatment. A message is generated using this information (for example, ‘It is advised to stop OAC and start antiplatelet drugs, due to the fact that this patient has a CHADS2 score of 1’). There are a total of 11 unique messages.

### Baseline measurements

Baseline measurements will be performed to determine the current proportion of patients with AF who are treated in accordance with the guideline. These data will be gathered by automatically evaluating the decision rule on all patients in an anonymized copy of the GP’s EHR databases before the start of the study.

### Regulatory aspects

The medical ethics committee of the AMC deemed this study exempt from ethical approval because the intervention is a way of providing valid medical information to GPs and does not change medical treatment or use invasive procedures. Furthermore, only anonymized patient data is used for analysis.

Every practice has provided written consent to participate in this study via a designated GP representative.

This trial is registered with the Dutch trial register under number 3570.

### Data collection

Whenever a message is shown, clicked, or ignored in the CDSS plugin, this event is stored in a database along with the current time and date. This will allow us to analyze the way the system is used by the GPs and will inform us about possible improvements that can be made. The responses GPs give to the questions about the type of stroke and the reason to ignore the message, if any, are saved inside the XML document and sent back to the remote server where the document is stored. During the study, these XML files will be opened and the responses will be extracted. To calculate adherence rates we use anonymized patient data (disease list, medication, GP contacts, lab data), which are extracted directly from the GP’s EHR databases.

### Population

We will include GP practices in the Netherlands that use the EHR for which the plugin was developed. Initially, we will include every GP practice (18 clusters of GPs, with a total of 35 GPs) that is part of the GAZO (a network of GP practices in Amsterdam Southeast). Decision support messages will be shown for every patient with atrial fibrillation (both new and existing patients) who visits the GP in his or her practice.

### Sample size and analysis

A power analysis showed that 300 patients are needed for a power of 0.8 when assuming a base adherence of 50%, an absolute effect size of 10% for intervention group 2 and 20% for intervention group 3, with a test significance level of 0.05. Because GPs are clustered for randomization, a correction for these clustering effects was introduced. For the cluster correction analysis, an intraclass correlation of .1 was assumed, based on studies using similar clustering [14]. This resulted in a required sample size of approximately 500 patients to result in an effective sample size of 300.

When data collection has been concluded, differences in adherence percentages will be calculated per GP cluster. A chi-square test will be used to compare the differences in the three groups.

### Outcomes

The primary outcome is the difference in the proportion of patients with AF, treated in adherence to the guideline between groups 1, 2 and 3.

The secondary outcomes are the reasons GPs provide for deviating from the guideline and the manner in which they respond to required justification (intervention 2).

## Discussion

This study will investigate the effectiveness of a decision support system in primary care by implementing a system that advises the general practitioner on antithrombotic treatment for stroke prevention in patients with atrial fibrillation. We will use a novel system that does not rely on pop-ups, but rather a sidebar that displays real-time messages concerning the actual patient, as studies have shown this to be the most effective timing for a CDSS [10].

The success of the study depends on the ability of the system to attract the attention of the GP and on the quality of data stored in the EHR. One of our challenges was finding the right mode of presentation, where messages are visible to the GP without interfering with their usual workspace. This meant we had to investigate computer use among GPs, especially which screen resolutions were used and how much space was available on their screen for our system. As it turned out, many GPs were working on older systems with low-resolution screens. We therefore had to adapt our system to actively show part of the message titles, the rest being entirely visible on demand. We hope this mode of presentation will not interfere significantly with the GP’s workspace, while remaining visible enough for the GP to notice.

### Guideline conversion

We could not fully convert the complete guideline into our decision rule, as some elements were not available as structured data in the GP’s EHR. For instance, the finding ‘Fundus bleeds of the eye’ has no code that can be used to identify it. Thus, we had to adapt our decision rule to work with the data that were available. Also, severe renal failure and liver failure were not defined with cut-off values, so we had to define these. These are important factors to consider during future guideline development, as decision support systems are expected to play an increasingly important role in healthcare. The recently introduced Logical Elements Rule Method (LERM) could play an important role in ensuring guideline-CDSS compatibility [15].

### Reasons for deviation from the guideline

Based on findings in literature on this subject, we expect GPs will have a number of valid reasons to provide treatment different from the guideline [8,16]. These are expected to be in the following categories: patient preference, diagnostic uncertainty and contextual factors (for example, medication prescribed by specialist). Almost all the other reasons we expect GPs to report will be related to incomplete registration. For instance, a contraindication for oral anticoagulation cannot be a reason to disregard the system’s advice, because the system checks for contraindications. Thus, the GP needs to update EHR to reflect the latest clinical situation.

### Effect size and reasons for non-adherence

We expect a 10% and 20% absolute effect size in intervention groups A and B, respectively. We hypothesize that the main reason for non-adherence is that there are too many parameters in this guideline to be evaluated effectively during a 10-minute visit. If a patient develops diabetes and reaches the age of 75, that has an impact on multiple guidelines. It is virtually impossible to know every guideline by heart, as there are currently one hundred Dutch GP guidelines, 10% of which are revised every year. Hence, the GP will often not be aware that the patient in fact needs to receive OAC instead of aspirin due to the fact that his or her CHADS2 score increased by two points.

## Conclusion

This paper describes the protocol for a cluster randomized trial to study the effects of a clinical decision support system in patients with atrial fibrillation. The system is characterized by a non-interruptive presentation and real-time messages that are updated after each action the GP performs (if the action is part of the decision rule). We expect an absolute effect size of 10 to 20%, because current guideline adherence is low (around 50%) and the guideline might be missed or is difficult to evaluate during a patient visit in a busy practice.

## Trial status

The CDSS plugin is currently being developed and tested. It is expected to be finished in March 2013, at which time a pilot period of one month will be started to identify any problems. After 1 month we will start collecting data.

## Abbreviations

AF: Atrial fibrillation; CDSS: Clinical decision support system; CRE: Clinical rule engine; EHRS: Electronic health records system; GP: General practitioner; ICPC: International Classification of Primary Care; OAC: Oral anticoagulant; XML: Extensible markup language.

## Competing interests

The authors declare they have no competing interests.

## Authors’ contributions

*DLA*: Converting the guideline to a decision rule, coordinating development with the involved parties, substantial programming for the plugin, writing this protocol. *HCPMW*: The original study protocol, acquiring funds, guiding the project, co-authoring the protocol, taking part in the justification committee. *AA*-*H*: The concept of the CDSS plugin, getting the necessary parties involved, guiding the project, co-authoring the protocol. *SE*: The concept of the CDSS plugin, working out the details of the CDSS plugin concept, coordinating with the involved parties. *HRB*: Co-authoring the protocol, taking part in the justification committee. *RJGP*: Co-authoring the protocol, taking part in the justification committee. All authors provided revisions to the paper, and all authors read and approved the final manuscript.
